# Histological Observation of Islet Hemorrhage Induced by Diagnostic Ultrasound with Contrast Agent in Rat Pancreas

**DOI:** 10.1371/journal.pone.0021617

**Published:** 2011-06-28

**Authors:** Douglas L. Miller, Chunyan Dou, Dorothy Sorenson, Ming Liu

**Affiliations:** University of Michigan Health System, Ann Arbor, Michigan, United States of America; Stanford University Medical Center, United States of America

## Abstract

Contrast enhanced diagnostic ultrasound CEDUS has been shown to induce capillary hemorrhage in heart and kidney. This study characterized the capillary hemorrhage induced in rat pancreas. The pancreata of anesthetized hairless rats were accessed by laparotomy. A 1.5 MHz diagnostic ultrasound probe with 2.3 MPa peak rarefactional pressure amplitude and 1 s intermittent trigger was used to scan the pancreas, located at the focus (3.8 cm), through saline coupling. The probe was swept to expose the entire organ in 5 min during infusion of Definity® contrast agent at 10 µL/kg/min, and this was repeated in a reverse sweep. The entire pancreas was removed, spread flat for fixation and histological slides were prepared from the mid-plane. Slides were scored blind for islet hemorrhage over the entire area of the organ. Intra-islet microlesions were evident and hemorrhage surrounded many islets. The hemorrhage often impacted nearby acini, and expanded into inter-lobular septa. In CEDUS pancreata removed soon after scanning, 76.2±11.8% (n = 6) of islets had evidence of hemorrhage and/or islet microlesions compared to 1.1±2.5% (n = 5) for sham CEDUS (P<0.001). In pancreata removed after 4 hr, fibrin formation was detected by immunohistology in the hemorrhage and intra-islet microlesions. Diagnostic ultrasound with contrast agent induced substantial capillary hemorrhage in rat pancreas, concentrated particularly in the islets.

## Introduction

Contrast agents have been developed to enhance diagnostic ultrasound images. These agents are injectable suspensions of gas bodies (stabilized microbubbles) which circulate with the blood and provide strong echos from normally poorly echogenic blood-filled regions. For example, Definity® (Perflutren Lipid Microsphere injectable suspension, Lantheus Medical Imaging, N. Billerica, MA) has 1.2 10^10^ gas bodies per mL with a mean diameter range of 1.1–3.3 µm. This agent is approved in the USA for use in patients with poor echocardiograms to opacify the left ventricular chamber and to improve the delineation of the left ventricular endocardial border. Contrast-enhanced diagnostic ultrasound (CEDUS) has many other potential applications, and is used in many countries for examination of liver, kidney, and other organs. CEDUS is not yet approved for use in pancreas imaging. The pancreas is an excellent candidate for diagnostic ultrasound examination, but can be somewhat difficult to image [1]. Because of this difficulty, CEDUS can be a valuable addition to the ultrasound examination [2], [3]. The approach used for contrast ultrasound in the pancreas can be transabdominal or endoscopic [4].

In CEDUS, the gas bodies are driven into radial oscillation by the ultrasound pulses and produce strong echos. The gas bodies can be destabilized and destroyed by the ultrasound pulses. This phenomenon forms the basis for perfusion re-fill imaging, in which the gas bodies in tissue are intermittently destroyed, typically using the highest output of the machine. Blood containing fresh gas bodies then is allowed to refill the tissue, demonstrating perfusion at the capillary level. The pulsation is a form of acoustic cavitation, which can yield high amplitude “inertial” cavitation even for a single pulse when gas bodies are destabilized. Cavitation generates mechanical perturbation in its vicinity and is a mechanism for bioeffects of ultrasound. CEDUS has been shown to induce capillary hemorrhage in heart, kidney and other tissues [5]. The pancreas may be susceptible to CEDUS related capillary injury, especially the islets. The islets constitute only about 2% of the pancreatic volume, but receive 10–20% of the blood flow to the entire organ [6]. Furthermore, the human pancreas contains only about 10^6^ islets, while a dose of contrast agent may contain billions of gas bodies (e. g. for Definity®, the bolus dose of 10 µL/kg has 8.4 billion gas bodies for a 70 kg patient). The purpose of this study was to histologically examine and characterized the possible occurrence of capillary hemorrhage in rat pancreas, particularly in islets, induced by CEDUS.

## Results

**Figure 1:**
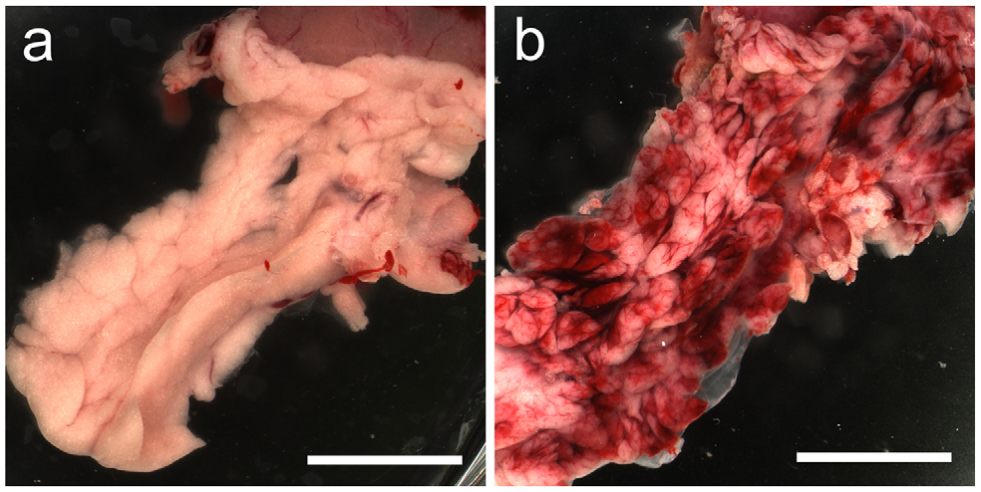
Macroscopic photographs of pancreata from (a) a sham CEDUS and (b) from a 2.3 MPa CEDUS without injected glucose. The CEDUS pancreas shows substantial hemorrhages visible in many of the lobes. Scale bars 1 cm.

**Figure 2:**
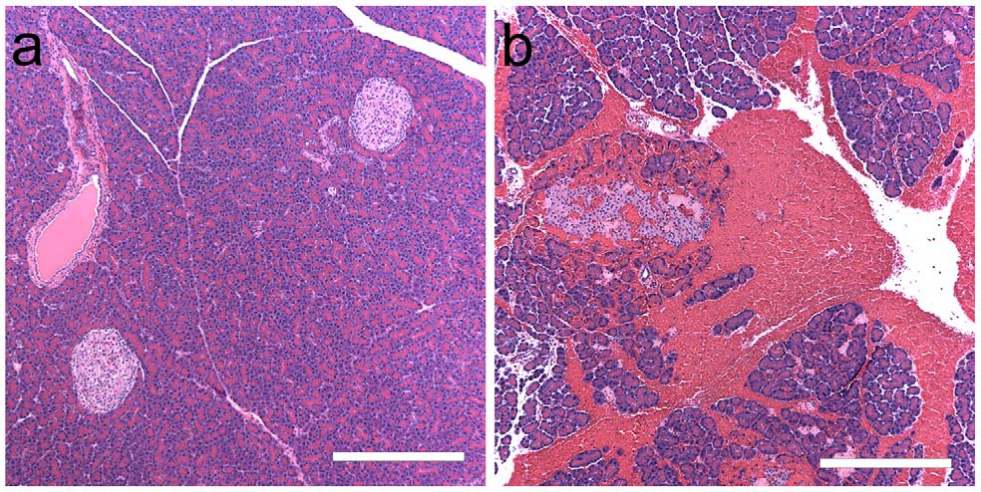
Wax histology with H&E stain from (a) a sham CEDUS and (b) a CEDUS pancreas without injected glucose. The CEDUS pancreas has an islet with a microlesion and hemorrhage extending into expanded interlobular septa. Scale bars 0.5 mm.

Each pancreas was photographed after trimming for histology. Figure 1 shows the appearance of a sham pancreas and a CEDUS pancreas at 2.3 MPa. The sham pancreas was creamy white in color with no evidence of sub-capsular hemorrhage. The CEDUS organ had visible evidence of sub-capsular hemorrhage, which was present in many lobules. The lobules were also more distinctly visible, possibly indicating edema. In histology, the sham samples appeared to be normal as shown in Fig. 2a. The 2.3 MPa CEDUS produced notable islet hemorrhage, which often surrounded the islet and extended into interlobular septa, as shown in Fig. 2b. There was little apparent difference between the pancreata from normal and glucose injected rats, although the macroscopic appearance of the hemorrhage in CEDUS samples seemed to be somewhat more copious.

**Figure 3:**
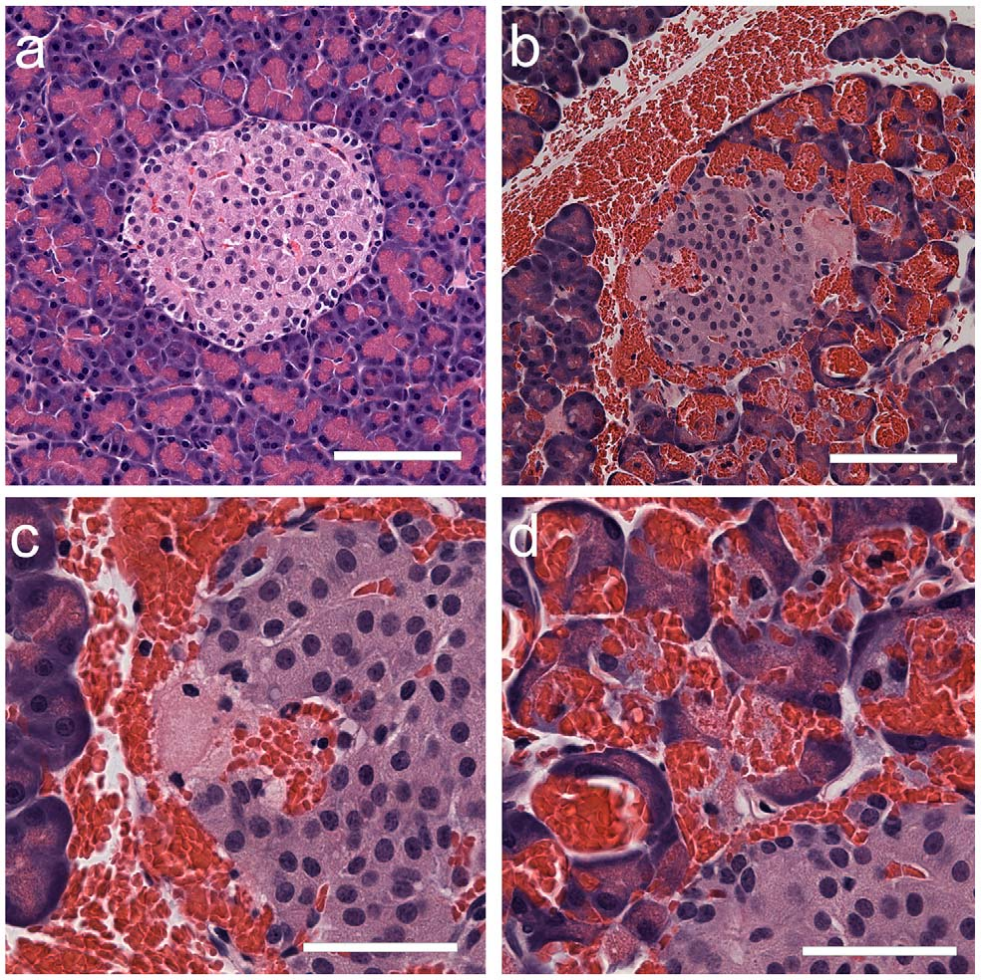
Wax histology with H&E stain from rats with glucose injection from (a) a sham CEDUS pancreas with a normal islet, and (b) from a CEDUS pancreas with a hemorrhaged islet. Scale bars 100 µm. The hemorrhaged islet had an apparent microlesion (c) with hemorrhage and an amorphous region of pink-stained material. In addition, acini around the islet (d) were impacted by the hemorrhage with erythrocytes apparently displacing the acinar cells. Scale bars 50 µm.

Upon closer histological examination, sham CEDUS islets appeared normal, while many CEDUS islets had intra-islet microlesions evident with hemorrhage as shown in Fig. 3. The microlesions, which were likely points of intra-islet hemorrhage, had an amorphous pink appearance with possible associated islet cell injury, as shown in Fig. 3c. The hemorrhage seemed to spread outward into the exocrine tissue, as shown in Fig. 3d. From this histology, the exact location of the erythrocytes in relation to the acinar cells was uncertain. In addition, some microlesions or hemorrhages were seen in the exocrine tissue, but these were difficult to quantify.

**Figure 4:**
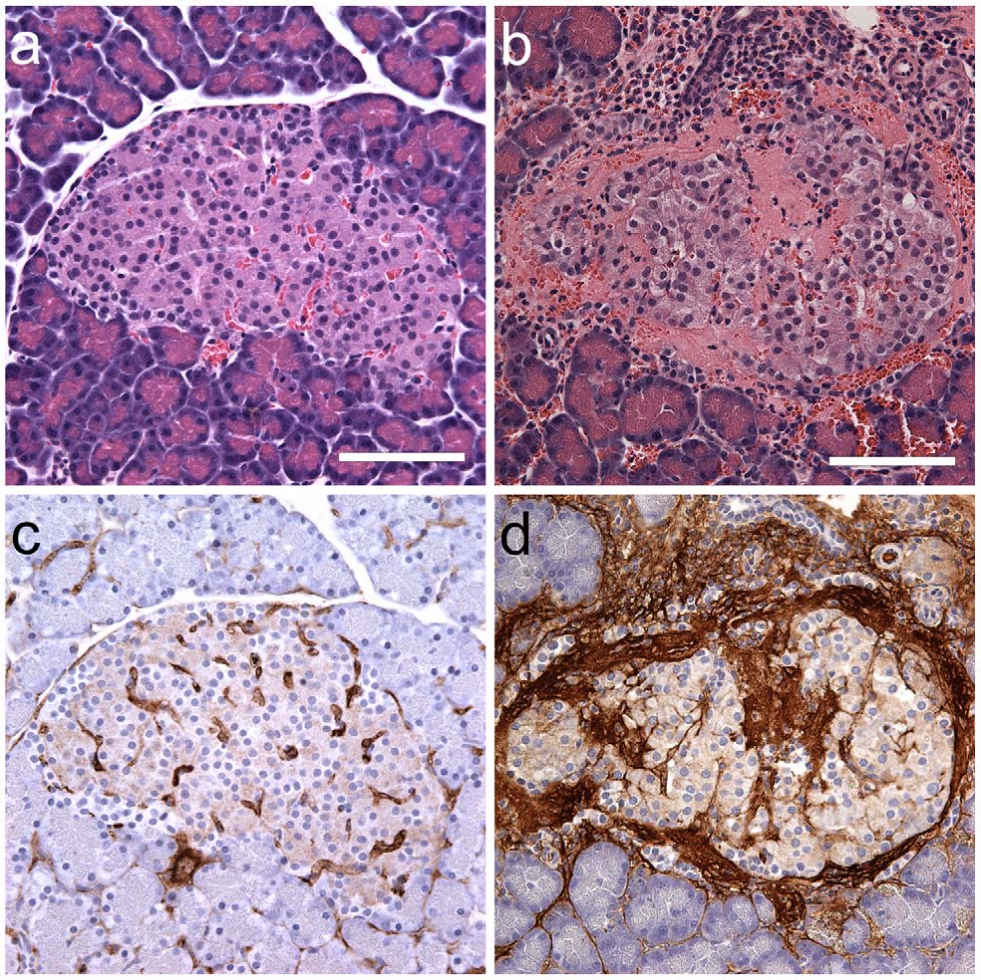
Histology with H&E staining of pancreata removed 4 h after (a) sham CEDUS or (b) CEDUS. The CEDUS islet (b) has hemorrhage and substantial areas of the amorphous pink material as in Fig. 3c. In addition, the CEDUS islet seems to have numerous additional cell nuclei, which are stained dark blue by the hematoxylin and may indicate early inflammatory infiltrating cells. In (c) the normal islet in (a) is shown with immunostaining for fibrin/fibrinogen, which stains residual plasma in capillaries brown. In (d) the hemorrhaged islet in (b) is shown with the immunostaining, which revealed substantial fibrin (clotting) associated with the amorphous pink material in (b). Scale bars 100 µm.

In some rats, sacrifice and pancreas removal were delayed 4 h. Examples of sham and CEDUS pancreata are shown in Fig. 4. Serial sections corresponding to the H&E stained sections in Fig. 4a and 4b were also stained for fibrin/fibrinogen to help identify regions with clotting of the hemorrhage. The sham islet (Fig. 4c) has some brown staining, which corresponds to the normal intra-islet capillaries due to fibrinogen in retained plasma. The CEDUS islet (Fig. 4d) has prominent brown stained areas corresponding to both the hemorrhage and intra-islet microlesions, which are shown in Fig. 4b. In addition, the 4 h CEDUS slides appeared to have extra cells (e. g. in comparison to the islet in Fig. 3b) indicated by the hematoxylin staining of cell nuclei. The extra cells were likely indicative early inflammatory cell infiltration in response to the injury and hemorrhage.

**Figure 5:**
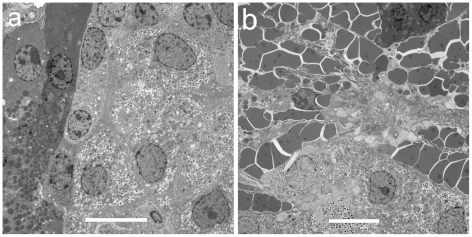
Transmission electron microscopy images of (a) a sham CEDUS and (b) an CEDUS pancreas with injected glucose. The images are at the periphery of islets, showing both islet cells and acinar cells. The tissue in (b) is perturbed by the hemorrhage as in Fig. 2b, which has scattered erythrocytes and evidence of clotting. Scale bars 10 µm.

**Figure 6:**
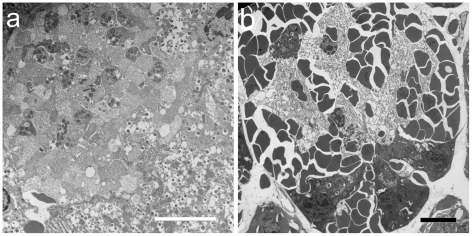
Transmission electron microscopy images of the types of microlesions seen in **Fig. 3c and 3d**. The amorphous material seen in Fig. 3c appears in TEM to be an aggregated mass of platelets (a), which likely formed to stem the hemorrhage (scale bar 5 µm). Erythrocytes impinging upon an acinus (b) appear to be mostly inserted between acinar cells, but some actually appear to be inside of disrupted acinar cells (scale bar 10 µm).

Transmission electron microscopy (TEM) images were made in an effort to characterize the intra-islet microlesions. Examples of sham and CEDUS samples are shown in Fig. 5. In the CEDUS samples, regions consisting of accumulations of platelets were evident as shown in Fig. 6a, which also includes apparently disrupted islet (probably beta) cells. These regions likely correspond to the intra-islet microlesions, which had an amorphous appearance, as in Fig. 3c. These were presumably the clot formations consequent to the hemorrhage. In Fig. 6b, acinar cells are shown with hemorrhage erythrocytes. Some of the clusters of erythrocytes appeared to be adjacent (i. e. between) the cells, while others apparently were actually inside the cells. These cells may have been injured by the hemorrhage impact, rather than directly by the cavitation activity.

**Figure 7:**
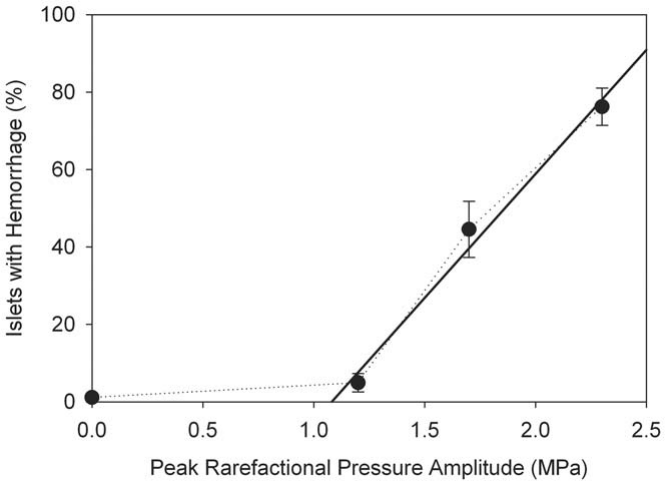
A plot of the percentage of islets with hemorrhage for the different CEDUS PRPAs used. The data from the three CEDUS groups are fitted with a linear regression line, which indicated a threshold at about 1.1 MPa.

The percentage of islets with hemorrhage decreased with decreasing peak rarefactional pressure amplitude (PRPA), as shown in Fig. 7. The results for 1.6 and 2.3 MPa CEDUS were significantly different from the sham (P<0.001). The result for 1.2 MPa CEDUS was not statistically significantly different from the sham, although islet hemorrhage was found in 3 of 5 pancreata in this group. This condition may therefore be near the PRPA threshold for this effect. The solid line in Fig. 7 is a linear regression on the three CEDUS points (r^2^ = 0.87), for which the line crosses zero islet hemorrhage at about 1.1 MPa (equivalent Mechanical Index = 0.9). The results for the 2.3 MPa CEDUS groups with glucose injection and with delayed pancreas removal were both significantly increased (P<0.001) relative to their respective shams (both zero). The islet hemorrhage results for the three 2.3 MPa CEDUS groups were 76.2%±11.8% for normal group, 77.9%±20.5% for the 4 h delay group, and 85.9%±12.7% for the glucose injection group, with no significant differences.

Blood glucose was measured before and after treatment. There were no significant trends for CEDUS with increasing MPa, relative to shams. Overall, for 30 rats, the before-test result was 81.4±11.8 mg/dL, and the after-test result was 107.4±12.6 mg/dL. For the 12 rats with injected glucose the before-test result was 76.7±11.5 mg/dL, and the reading 15 min later was 410.8±14.8 mg/dL. In addition, blood insulin was measured in plasma for 4 sham and 4 CEDUS rats before and after treatment. The results are presented in Table 1. The before and after results for the shams were not significantly different for a paired t-test, while the results for CEDUS were significantly different (P<0.05), which indicates that the injury from the CEDUS produced an increase in blood insulin.

**Table 1:** Results of the blood insulin tests with significance evaluated using a paired t-test.

Condition	Before	After	P value
Sham	0.21±0.24	0.62±0.31	NS
CEDUS	0.34±0.25	1.6±0.98	<0.05

Values are given in ng/mL in plasma.

## Discussion

This study characterized the capillary hemorrhage induced in rat pancreas, particularly in islets, by CEDUS. A diagnostic ultrasound probe was swept to expose the entire organ in 5 min during infusion of Definity® contrast agent at 10 µL/kg/min, and this was repeated in a reverse sweep. The entire pancreas was fixed for histology and slides were scored blind for islet hemorrhage over the entire area of the organ. Islet hemorrhage was noted, which often extended into expanded interlobular septa. In CEDUS pancreata removed soon after scanning at 2.3 MPa, 76.2±11.8% (n = 6) of islets had evidence of hemorrhage and/or islet injury compared to 1.1±2.5% (n = 5) in shams (P<0.001). A threshold for the effect was identified at about 1.1 MPa. This threshold was higher than the threshold of 0.73 MPa found for glomerular capillary hemorrhage in rat kidney [7], which was estimated using a more sensitive method. Pre-treatment injection of glucose, which increases islet blood flow, gave a small increase in islet hemorrhage but this was not statistically significant. It should be noted that nearly all the islets had evidence of hemorrhage both with and without glucose. The injected glucose might have produced a greater difference in islet hemorrhage for lower (e. g. 1.6 MPa) exposures.

Intra-islet microlesions were evident in many islets with hemorrhage (Fig. 3c) and the material located at the injury sites appeared to be aggregated platelets upon examination by TEM (Fig. 6a). The circulation in the rat pancreas is such that the islets receive 10–20% of the blood flow to the entire organ [6] and the blood tends to flow outward from the interior of the islet [8]. Given this pattern, the initial hemorrhages likely occurred inside the islet (rather than in the exocrine tissue) with hemorrhage flowing outward and into the exocrine tissue. Fibrin formation was detected by immunohistology in the hemorrhage and intra-islet microlesions in samples removed 4 h post-CEDUS (Fig. 4d). In the exocrine tissue, acini adjacent to the hemorrhaged islet appeared to be injured by the hemorrhage flow (Fig. 3b). In TEM, erythrocytes were located between acinar cells in most cases (Fig. 5b), but appeared to be located inside some evidently disrupted acinar cells (Fig. 6b). Disruption of acinar cells can be a factor in acute pancreatitis [9], for which enzyme release can lead to further injury of nearby cells.

With ultrasonic cavitation occurring inside the islets, the injury of beta cells is possible. In an effort to detect perturbation of the beta cells, blood insulin was measured in plasma samples (Table 1). A small differential increase in insulin after CEDUS was detected. In further studies, the use of more rapid blood samples, even during exposure, might be of value for characterizing this effect, owing to the rapid turnover time (half life about 4 min) of insulin in the blood [10].

The intra-islet hemorrhage induced by CEDUS appeared to be a unique mode of pancreas injury. This basic research in non-ionizing radiation biology represents a contribution to the newly developing topic of ultrasonic cavitation biology. The precise control and targeting of this phenomenon by manipulating the contrast agent dose and ultrasound exposure may afford the investigator new opportunities for research. Islet hemorrhage, followed by inflammation and progressive fibrosis, has been found to occur with age in spontaneously diabetic rats [11]. This spontaneous hemorrhage and fibrosis can lead to reduced islet function [12]. In a rat model of type 2 diabetes, disruption of endothelium and intra islet hemorrhage has been observed as the disease progresses to islet failure [13]. The production of islet hemorrhage at will by CEDUS-induced cavitation may therefore provide a unique means to model early diabetic changes in laboratory animals. In addition, this phenomenon may present opportunities for therapeutic treatment. For example, Chen et al. [14] have reported progress in targeting ultrasound mediated gene therapy to the pancreatic islets by this method.

Diagnostic ultrasound with a commercial ultrasound contrast agent induced significant islet hemorrhage in rat pancreas. In this histological study, a high level of effects was sought to provide abundant material for detailed observations. For clinical CEDUS, the amount of islet hemorrhage, which might be expected based on these results, is uncertain. Nevertheless, to minimize the risk of islet hemorrhage, the ALARA (As Low As Reasonably Achievable) safety principle should be practiced by minimizing high MPa microbubble destruction in contrast-enhanced diagnostic examinations of the pancreas.

## Methods

### Ethics statement

All in vivo animal procedures were conducted under approval number 07875 of the University of Michigan University Committee on Use and Care of Animals.

### Animal preparation

Hairless rats (CD strain, Charles River, Wilmington, MA, USA) were obtained at 8–10 weeks of age and weighed 317±36 gm. After over-night fasting, the rats were anesthetized by intraperitoneal (IP) injection of sodium pentobarbital (Nembutal, Lundbeck Inc. Deerfield IL USA) at 50 mg/kg. A 24 gauge cannula was inserted into a tail vein for IV injections, and a small rectal thermometer was used for temperature monitoring. For some rats, a jugular vein catheter was also inserted for blood sampling. A midline laparotomy incision was made to allow open access to the pancreas. The rats were placed in a dorsal recumbent position between two supports on a heated pad. The sides of the incision were then sutured to the supports to maintain the open abdomen, and the intestines and stomach were moved to allow direct visualization and scanning of the entire pancreas. Warmed saline was added to the abdominal cavity to acoustically couple a bag of warm saline to the pancreas. The 6 cm wide bag was positioned with a ring stand and contained about 200 mL of saline. The rat was otherwise covered with gauze sponges to help maintain body temperature, which averaged 36.9±0.5°C. Forty five rats were used, with two lost from the study, apparently due to anesthetic death.

A blood sample was tested for blood glucose before and after treatment using a blood glucose meter (Accu-Chek Compact Plus, Roche Diagnostics, Indianapolis IN USA). Some rats received an IV injection of 2 g/kg glucose in a 50% solution before treatment, and for these rats the second glucose test was timed to be 15 min after the glucose injection. The glucose injection was intended to increase the pancreas and islet blood flow [15]. Rats with the jugular vein catheter had blood samples withdrawn before and after treatment for measurement of blood insulin using a rat insulin ELISA kit on plasma samples (Cat. no. 90060, Crystal Chem Inc. Downers Grove IL USA).

### Ultrasound

The diagnostic ultrasound machine was a Vingmed System Five (General Electric Co., Cincinnati OH) with FPA2.5 phased array probe, which was protected with a probe cover (Cone Instruments, Solon OH). This probe was clamped in an adjustable support stand and aimed downward at the pancreas with the probe partly immersed in the saline in the bag. The distance from probe to pancreas was adjusted to place the organ in the focal zone 3.5–4.5 cm from the transducer face. The machine was set to 1.5 MHz, 5 cm focus, 10 cm depth and triggered imaging with a 1 s interval between dual scans (50 ms apart). Dual scans were employed to confirm contrast destruction by the loss of enhanced contrast in the second image. The peak rarefactional pressure amplitude (PRPA) of the 1.5 µs pulses (about 2 cycles) was measured in a water bath with a calibrated hydrophone (model 805, Sonora Medical Systems Inc., Longmont CO USA). At the maximum (0 dB) setting, the PRPA was 2.3 MPa with an equivalent Mechanical Index (eMI) of 1.9, which was essentially at the upper limit for diagnostic ultrasound. Some rats were scanned at power settings of −3 dB or −6 dB which gave PRPAs of 1.6 MPa (eMI = 1.3) and 1.2 MPa (eMI = 0.98), respectively. The scan plane thickness (i. e., the −6 dB beam width) was 4.3 mm.

The probe was swept 25 mm to expose the entire organ in 5 min during infusion of diluted Definity® contrast agent. This was repeated in a reverse sweep with freshly diluted contrast agent. Initially, the sweeping movement was performed manually using a slide with micrometer, but this was replaced with a motorized positioning system (Xslide, Velmex Inc. Bloomfield NY USA) to provide a uniform rate of sweep. A new vial of Definity® was prepared each day according to the manufacturer's instructions. For infusion, 0.6 ml of Definity® was diluted in 3 ml of sterile saline in a syringe, which was connected to the tail vein cannula with an extension tube. The diluted agent was infused at 0.5 ml/kg/min with a syringe pump (Aladdin 1000, World Precision Instruments, Sarasota FL USA), which delivered 10 µL/kg/min of the agent. The circulating dose in rats during infusion is reduced greatly due to substantial gas-body destruction in the small animals [16]; therefore, the corresponding equivalent human infusion dose is uncertain (i. e. possibly substantially less). Sham CEDUS included the swept ultrasound scanning with out contrast agent infusion, followed by infusion of the contrast agent with out ultrasound. The swept-scanning differed from previous research in kidney, for which the scan plane was stationary for a shorter exposure duration (e. g. 1 min during 10 µL/kg/min infusion [7]. This whole-organ exposure was performed in this study to include more exposed islets in the resulting histological sections.

### Histology

After exposure, a large tissue sample was removed from the abdomen, including the pancreas together with the spleen, stomach, and duodenum. This sample then was trimmed to include the pancreas and a small segment of duodenum and then spread flat for fixation in neutral buffered formalin. For some rats, removal of the organ was delayed 4 hr. The pancreata were cut approximately in half to yield a head and a tail sample for each pancreas. Histological slides were prepared from the mid-plane of each sample by the Research Histology and Immunoperoxidase Laboratory of the University of Michigan Comprehensive Cancer Center Tissue Core. Slides were scored blind for islet hemorrhage over the entire area of the organ. For 39 rats scored in this way, 32±13 and 48±14 islets were counted in the head and tail samples, respectively, for a total of 80±20 for the whole organ. The 4 hr samples were used to prepare serial sections, one with H&E staining and the next with immunohistochemistry staining with fibrin/fibrinogen antibody, as described previously for kidney [17].

### Transmission electron microscopy

For transmission electron microscopy (TEM), the organ was cut into ∼1 mm pieces and fixed in 2.5 per cent glutaraldehyde in 0.1 M Sorensen's buffer and post fixed in osmium tetroxide in the same buffer. The tissue was dehydrated in a graded series of ethanol, transitioned through propylene oxide, and embedded in Epon. Ultra-thin sections were post stained with uranyl acetate and lead citrate and imaged on a Philips CM 100 electron microscope. Images were recorded digitally, using a Hamamatsu ORCA-HR camera system that was operated using AMT software (Advanced Microscopy Techniques Corp., Danvers, MA). The processing and TEM were performed at the Microscopy and Image Analysis Laboratory of the University of Michigan Department of Cell and Developmental Biology.

### Experimental plan and statistics

Eight groups of rats received different treatments. Groups of 5 rats had sham, 1.2 MPa, and 1.6 MPa CEDUS, and one group of 6 rats had 2.3 MPa exposure. Groups of 5 rats with glucose injection had sham and 2.3 MPa CEDUS. Groups of 4 rats with sham and 2.3 MPa CEDUS had a 4 hr delay to sample removal. One pancreas, from a rat which died at anesthesia, was used for a normal control sample. Finally, single rats with sham or 2.3 MPa CEDUS, without or with glucose injection, were used for TEM samples.

Results are provided as general observations common to different groups. In addition, results for islet hemorrhage scoring or biochemical measurements are given as the mean and standard deviation, or plotted as the mean with standard error bars. Statistical comparisons were made using the Student's t-test or Mann-Whitney rank sum test as appropriate.
